# Microperimetry and Optical Coherence Tomography Changes in Type-1 Diabetes Mellitus without Retinopathy

**DOI:** 10.3390/diagnostics11010136

**Published:** 2021-01-16

**Authors:** Elvira Orduna-Hospital, Judit Otero-Rodríguez, Lorena Perdices, Ana Sánchez-Cano, Ana Boned-Murillo, Javier Acha, Isabel Pinilla

**Affiliations:** 1 Aragon Institute for Health Research (IIS Aragon), 50009 Zaragoza, Spain; elvisabi14@hotmail.com (E.O.-H.); lperdices@gmail.com (L.P.); anaisa@unizar.es (A.S.-C.); anabomu@hotmail.com (A.B.-M.); j.acha.perez@gmail.com (J.A.); 2 Department of Applied Physics, University of Zaragoza, 50009 Zaragoza, Spain; juditaotro97@gmail.com; 3 Department of Ophthalmology, Lozano Blesa University Hospital, 50009 Zaragoza, Spain; 4 Department of Endocrinology, Miguel Servet University Hospital, 50009 Zaragoza, Spain

**Keywords:** diabetic retinopathy, total retinal thickness, inner retinal layer thickness, ophthalmology, macular sensitivity, microperimetry, swept source optical coherence tomography (SS-OCT), type 1 diabetes mellitus

## Abstract

Background: We aimed to measure and correlate inner retinal layer (IRL) thickness and macular sensitivity by optical coherence tomography (OCT) and by microperimetry, respectively, in type 1 diabetes mellitus patients (DM1) without diabetic retinopathy (DR). Methods: Fifty-one DM1 patients and 81 age-matched healthy subjects underwent measurement of the axial length (AL), retinal thickness in the macular ETDRS areas by swept source (SS)-OCT and macular sensitivity by microperimeter. Results: The total retinal and IRL thicknesses were thicker in the DM1 group (*p* < 0.05) in practically all ETDRS areas, and they had a generalized decrease in sensitivity (*p* < 0.05) in 9 areas between both groups. There was a significant negative correlation between retinal sensitivity and age in all areas and in visual acuity (VA) in 5 out of the 9 areas for DM1 patients. Only a mild negative correlation was observed between retinal sensitivity in the 5° nasal inner (5NI) area and in IRL thickness in the temporal inner (TI) area (−0.309 with *p* = 0.029) in the DM1 group. Conclusion: Aging and disease evolution in DM1 patients without DR signs generate a decrease in retinal sensitivity. There was a direct relationship between retinal sensitivity and macular thickness in the DM1 group.

## 1. Introduction

Although diabetic retinopathy (DR) is regarded mainly as a microvascular disorder [1], there is evidence that supports the appearance of functional changes in early stages [2,3]. It suggests that prior to the appearance of DR, there is neurodegeneration that causes functional [4,5] and structural abnormalities [6,7], such as a decrease in retinal sensitivity detectable by microperimetry [8,9,10] or changes in macular thickness seen on optical coherence tomography (OCT) [11,12].

In diabetes, there is evidence of increased retinal cell apoptosis [13], which begins affecting the ganglion cell (GC) bodies with shrinkage of dendritic arbors and their consequent axonal involvement [14]. Neuronal changes can be detected measuring the different inner retinal layers (IRL), such as the retinal nerve fiber layer (RNFL), the GC layer (GCL) and the inner plexiform layer (IPL), known as the ganglion cell complex (GCC), in addition to the total retinal thickness by OCT. Previous studies have demonstrated changes in the total retinal thickness and IRL thickness in diabetic patients without DR [11,15,16,17,18].

Microperimetry shows decreased macular sensitivity despite high visual acuity (VA) in diabetic patients without DR signs [8,9,10], suggesting that microperimetry may give additional information reflecting functional impairment in diabetic patients before vascular manifestations occur.

Many studies indicate early neurodegeneration in diabetes in terms of functional and morphological impairments, but only a few of them have correlated OCT changes with retinal sensitivity results [19,20]. Diabetic subjects with no or minimal vascular signs have been evaluated by spectral domain (SD)-OCT and by microperimetry, but only mean foveal sensitivity and foveal thickness measurements have been analyzed. Moreover, to our knowledge, the correlation between the IRL and retinal sensitivity of the macula was only investigated by Montesano et al. [8].

The purpose of this study was to measure the total retinal thickness and the different IRL thicknesses by swept source (SS)-OCT and macular sensitivity by microperimetry in type 1 diabetes mellitus (DM1) patients without diabetic retinopathy (DR) and to compare the results with those of healthy subjects. In addition, we studied the correlation of the structural and functional data obtained from the ETDRS macular grid.

## 2. Materials and Methods

This study was developed following the principles established in the Declaration of Helsinki and after the approval of the Ethics Committee for Clinical Research of Aragon (CEICA 18/2017), approved on 17 October 2017. Written informed consent was explained and signed by each participant before carrying out any type of exploration, giving them the freedom to leave the study at any time.

It is a prospective observational study on macular retinal thickness and sensitivity in 51 DM1 patients without DR signs with good glycemic control and in 81 age-matched healthy subjects from October 2018–October 2019. All the patients were evaluated at the Ophthalmology Department of the Lozano Blesa University Hospital by the same investigator.

The DM1 patients participating in the study had at least 9 years of disease evolution with no signs of DR identified by biomicroscopy, retinography or structural OCT. They were well controlled by the endocrinology unit. Blood samples were analyzed every six months. Glycosylated hemoglobin (HbA1c), lipid values and arterial blood pressure were maintained under extreme control.

The inclusion criteria were a VA over 20/25 on the Snellen chart to avoid difficulties in performing ophthalmic tests, refractive errors between +5.00 to −5.00 spherical diopters and less than 3 diopters of astigmatism, a normal anterior segment examination with slit-lamp biomicroscopy, intraocular pressure (IOP) less than 20 mmHg measured by Goldman tonometry and no fundoscopy anomalies observed by ophthalmoscopy, retinography or structural OCT. A detailed familial, systemic and ophthalmological medical history was obtained from each patient.

The exclusion criteria were the presence of any sign or manifestation of DR, glaucoma, optic nerve pathology, ocular inflammation or previous ocular surgery or procedure including laser therapy, ocular traumatism, anterior segment pathology or media opacification. Patients with other systemic pathology or neurodegenerative disease (Alzheimer’s, Parkinson’s, multiple sclerosis) were also excluded.

VA was measured by the Snellen chart and by the ETDRS 100% contrast test at four meters in LogMAR scale. The axial length (AL) was measured with the optical biometry IOLMaster^®^ 500 (Carl Zeiss Meditec, Oberkochen, Germany).

**Figure 1:**
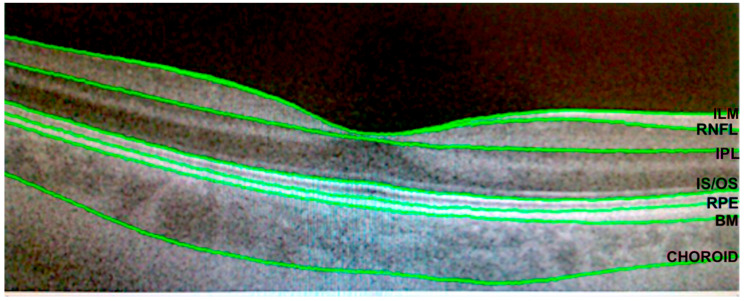
Segmentation of the different retinal layers as well as the choroid with the DRI Triton swept source optical coherence tomography (SS-OCT). The provided lines are as follows: ILM, inner limiting membrane line; RNFL, retinal nerve fiber layer line; IPL, inner plexiform layer line, IS/OS, inner/outer segments line; RPE, retinal pigment epithelium line; and choroid line.

Each participant was imaged by Deep Range Imaging (DRI)-Triton SS-OCT (Topcon Corporation, Tokyo, Japan) obtaining a macular 6.0 × 6.0 mm three-dimensional scan; automatic segmentation of each retinal layer was made with IMAGEnet 6 Version software 1.22.1.14101© 2014 Topcon Corporation (Figure 1) to evaluate the structural function of the retina in 3D [21].

The DRI Triton SS-OCT in its 3D macula protocol was used to obtain tomographic macular images and the image quality scale, which ranges from 0 to 100, must be over 60 [22].

**Figure 2:**
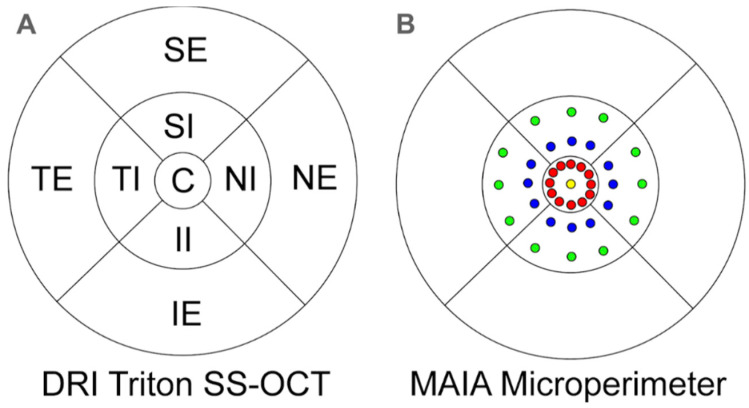
(**A**) ETDRS grid of a right eye divided into nine areas provided by the Deep Range Imaging (DRI) Triton SS-OCT. Four areas for the 6-mm perifoveal ring: nasal external (NE), temporal external (TE), superior external (SE) and inferior external (IE) areas; four areas for the 3 mm parafoveal ring: nasal internal (NI), temporal internal (TI), superior internal (SI) and inferior internal (II) areas; and the 1-mm central area. (**B**) The 37 sensitivity points provided by the Macular Integrity Assessment (MAIA) microperimeter and its location: one in the center (yellow), 12 in the 1° radius (red), 12 in the 3° radius (blue) and 12 in the 5° radius (green). One degree is equivalent to a radius of 0.3 mm; 3° to a radius of 0.9 mm; and 5° to a circle with a 1.5-mm radius. The center point and the 1° sensitivity points (0.6-mm diameters) correspond to the ETDRS 1-mm diameter center ring, and the localized sensitivity points at 3 and 5° (diameters of 1.8 and 3 mm, respectively) to the 3-mm diameter parafoveal ring of the ETDRS grid.

The 3D macula protocol measures in the 9 ETDRS areas [23], providing a circular macular map analysis divided in three concentric circles with diameters of 1, 3 (internal) and 6 (external) mm. The acquired values were the central or subfoveal area (1 mm, C), 3-mm parafoveal ring with four areas, temporal internal (TI), superior internal (SI), nasal internal (NI) and inferior internal (II) areas, and four other areas belonging to the 6-mm perifoveal ring including the temporal external (TE), superior external (SE), nasal external (NE) and inferior external (IE) areas to study each macular layer thickness (Figure 2). To evaluate the different layers, the DRI Triton SS-OCT software uses the refringent properties of each retinal layer.

In our study, we included 3 retinal protocols: the total retinal thickness from the ILM line to the BM line, called “retina” by the device; the “GCL+” formed by the GCL and the inner plexiform layer (IPL) (GCL-IPL complex) from the RNFL upper line to the IPL bottom line; and the “GCL++” or GCC, referring to the sum of the RNFL, GCL and IPL from the ILM line to the IPL line.

To evaluate the macular sensitivity and functional integrity we used Macular Integrity Assessment (MAIA, Macular Integrity Assessment system; Topcon Corporation, Tokyo, Japan) microperimeter in each participant in scotopic conditions and without pupillary dilation. The MAIA is designed to identify any decrease sensitivity compared to the normal sensitivity for the patient’s age and to differentiate pathological changes associated with retinal diseases. This instrument automatically calculates a macular integrity estimation and an analysis for the residual MS threshold values in dB, classifying macular integrity as normal (loss of sensitivity less than 40%), suspicious (loss of sensitivity from 40% to 60%) and out of normal limits (sensitivity losses greater than 60%) [24]. The MAIA software version was 1.6.3.

During the first 10 s of testing, the instrument calculates the location of the “preferred retinal locus” (PRL), which serves as an alignment reference, locating the foveola when the patient looks at the fixation point without stimuli yet projected (PRL-High). Then, a second estimate of this location is taken at the end of the test, which is the reference point for the other points (PRL-Low). Both estimated data are graphically represented on the screen; the MAIA classifies the fixing stability based on the location of PRL-Low as follows: stable, more than two-thirds of the attachment points are centered within the PRL-Low circle; relatively unstable, more than one-third of the attachment points are centered outside the PRL-Low circle; and unstable, less than one-third of the attachment points are centered within the PRL-Low circle.

The MAIA sensitivity points were directly correlated to the same areas of the ETDRS grid provided by DRI-Triton SS-OCT. The MAIA provides 37 sensitivity points including one in the center, 12 in the 1° radius (corresponding to a radius of 0.3 mm), 12 in the 3° radius (corresponding to a circle with a 0.9-mm radius) and 12 in the 5° radius that represents a circle with a radius of 1.5 mm. Figure 2 represents the location of the different points and the ETDRS area in an emmetropic eye.

Thus, the mean of the retinal sensitivity thresholds calculated by the MAIA for the central and the inner rings were arranged in the central 1-mm ETDRS ring (13 sensitivity points) and the 3 and 5° radius MAIA thresholds corresponded to the SI, NI, II and TI areas of the 3-mm parafoveal ETDRS ring (mean of 6 sensitivity points per area).

With the MAIA, we evaluated the following data: fixation loss, retinal sensitivity, macular integrity index, mean total threshold, fixation stability (p1 and p2, percentage of fixation points with respect to the total within circles with 1° and 2° radii, respectively, classified as stable (p1 > 75%), relatively unstable (p1 < 75% and p2 > 75%) or unstable (p2 < 75%)), bivariate contour ellipse area (BCEA) where the fixation points are contained, BCEA63 (minor ellipse) and BCEA95 (major ellipse).

All the eyes included in the study had foveal fixation that was checked manually. Fundoscopic images were extracted and subsequently imported into SS-OCT for analysis and comparison.

The studied variables were recorded in an Excel database (Microsoft^®^ Office Excel 2011, Microsoft Corporation). Statistical analysis was performed with the Statistical Package for the Social Sciences (SPSS 24.0 Inc., IBM Corporation, Armonk, NY, USA).

First, a descriptive statistical analysis of the sample was carried out according to the demographic variables and clinical characteristics, and the mean and standard deviation of the continuous quantitative descriptive variables were calculated, while for the qualitative variables, the absolute frequencies and the corresponding percentages were obtained.

The distribution of normality of all the variables was also assessed with the Kolmogorov–Smirnov test. As they did not conform to normality, the non-parametric Mann–Whitney U test was used for the two independent samples (DM1 group and control group) and Bonferroni correction for multiple comparison was applied considering statistical significant differences with a value of *p* < 0.05.

Finally, with the Spearman correlation test, we correlated the anatomical and functional data of the DM1 patients.

## 3. Results

A total of 132 eyes were analyzed, of which 51 belonged to 51 DM1 patients (46.2% women and 53.8% men) with an average disease evolution of 25.88 ± 8.42 years and without DR signs, while 81 eyes corresponded to 81 healthy subjects (54% women and 46% men) without statistically significant differences between the gender (*p* = 0.371). The eye of each participant studied was randomly chosen.

The mean age in healthy controls was 42.41 ± 13.24 years (25 to 68) and the mean age in DM1 patients was 41.52 ± 13.21 years (24 to 68), without differences between groups (*p* = 0.361).

There were no differences (*p* > 0.05) between groups in mean spherical equivalent (SE), axial length (AL), IOP or LogMAR VA (Table 1).

### 3.1. Comparative Analysis of the Retinal Thickness

**Figure 3:**
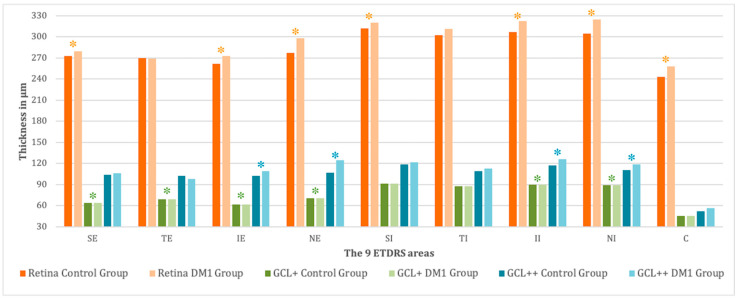
Mean thickness of the total retina (from the ILM line to the BM line), GCL+ (from the RNFL line to the IPL line) and GCL++ (from the ILM line to the IPL line) in the 9 ETDRS areas obtained by the DRI-Triton OCT for the control and the DM1 groups. Statistically significant differences between groups (*p* < 0.05) are marked with *. The measures are divided into 9 ETDRS areas. SE, superior external; TE, temporal external; IE, inferior externa; NE, nasal external; SI, superior internal; TI, temporal internal; II, inferior internal; NI, nasal internal; and C, central. Thickness values are expressed as μm.

We found differences between groups in the total retinal thickness measured by DRI-Triton OCT (Figure 3), except for the TE (270.06 ± 22.36 μm vs. 269.38 ± 17.96 μm, control vs. DM1 group, *p* = 0.701) and TI area (302.56 ± 23.97 μm vs. 311.46 ± 24.45 μm, control vs. DM1 group, *p* = 0.100). DM1 patients had a higher retinal thickness than healthy controls in practically all ETDRS areas, with the exception of the TE area.

Analyzing the GCL+ (Figure 3), there were no differences between both groups in the SI area (91.38 ± 8.52 μm vs. 94.25 ± 13.34 μm, control vs. DM1 group, *p* = 0.073), TI area (87.87 ± 8.83 μm vs. 89.94 ± 13.58 μm, control vs. DM1 group, *p* = 0.231) and the central area (45.54 ± 14.03 μm vs. 50.52 ± 19.19 μm, control vs. DM1 group, *p* = 0.109) that achieved significant differences in the total retinal thickness evaluation. We observed that the TI area was maintained without differences between groups.

Evaluating the GCL++ (Figure 3), the areas that were different between groups decreased, showing differences only in the nasal and inferior areas (IE: 102.11 ± 10.62 μm vs. 109.29 ± 18.72 μm, *p* = 0.028; NE: 106.92 ± 16.90 μm vs. 124.44 ± 15.76 μm, *p* < 0.001; II: 117.45 ± 11.87 μm vs. 126.38 ± 25.44 μm, *p* = 0.019 and NI: 110.61 ± 9.56 μm vs. 118.98 ± 17.10 μm, *p* < 0.001 in the control vs. DM1 group). Furthermore, the thickest pattern continued in the DM1 group, except for the TE area (102.45 ± 16.76 μm vs. 97.75 ± 12.12 μm, control vs. DM1 group, *p* = 0.142).

### 3.2. Comparative Analysis of Retinal Sensitivity

**Figure 4:**
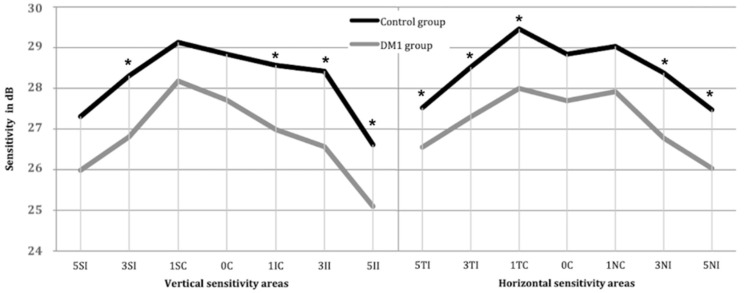
Mean retinal sensitivity in dB measured by the MAIA microperimeter in the ETDRS areas corresponding to the 3-mm parafoveal ring divided into 2 subrings (mean of 3 points per area at 5° and 3°) and the 1-mm central ring divided into 2 subrings (mean of 3 points per area at 1° and a central point): 5SI, 5° superior internal; 5TI, 5° temporal internal; 5II, 5° inferior internal; 5NI, 5° nasal internal; 3SI, 3° superior internal; 3TI, 3° temporal internal; 3II, 3° inferior internal; 3NI, 3° nasal internal; 1SC, 1° superior central; 1TC, 1° temporal central; 1IC, 1° inferior central; 1NC, 1° nasal central; and 0C, 0° central. Statistically significant differences are at *p* < 0.05 and are marked with *.

Analyzing the data obtained by the MAIA (Figure 4), we observed a generalized decrease in sensitivity in the DM1 group compared to the control group. In addition, there were 9 areas with differences between both groups (*p* < 0.05): in the 5° radius, the areas were the II, TI and NI; in the 3° radius, the areas were the SI, II, TI and NI; and in the 1° radius, the areas were the SC and TC. The sensitivities of the different areas are shown in Figure 4.

The rest of the parameters obtained by the MAIA are represented in Table 2. We only found differences in two of the analyzed parameters: the average threshold (28.16 ± 1.46 dB vs. 26.44 ± 3.47 dB, control vs. DM1 group, *p* = 0.005) and the fixation losses (0.42 ± 2.83% vs. 6.56 ± 12.99%, control vs. DM1 group, *p* = 0.013).

### 3.3. Anatomo-Functional Correlation Study

To correlate the structural and functional data obtained by DRI Triton SS-OCT and the MAIA, we used the Spearman test.

Regarding age, there was a significant negative correlation (*p* < 0.05) with retinal sensitivity in all areas in DM1 patients and with VA in 2 areas of the 5° ring: 5SI (−0.356 with *p* = 0.011) and 5II (−0.301 with *p* < 0.034); and in 3 areas of the 3° ring: 3SI (−0.341 with *p* = 0.015), 3TI (−0.369 with *p* = 0.008) and 3II (−0.341 with *p* = 0.015). There was no correlation between the sensitivity of the MAIA microperimeter and AL (Table 3).

Table 4 shows the correlation between the thickness values in the central circle of the ETDRS grid and the sensitivity values of the internal and central rings belonging to the DM1 group. We did not find any correlation.

The correlations between the thicknesses of the 3 protocols in the four parafoveal inner ETDRS areas (SI, TI, II and NI) with the measured thresholds of macular sensitivity in the 3 and 5° radii are also analyzed in Table 4. Only a mild negative correlation was observed in the GCL++ protocol between retinal sensitivity in the 5NI area and thickness in the TI area (−0.309 with *p* = 0.029).

## 4. Discussion

In this study, we found higher macular thicknesses in the DM1 group. It could reflect changes in the blood–retinal barrier, allowing fluid to enter the extravascular space, thereby leading to retinal thickening in the macular area. Other possibilities that have been described include modifications in Müller cells or an increase in the height of RPE cells [11,25].

The GCL+ and GCL++ (Figure 3) showed increased thickness at the macular level in DM1 patients. The GC bodies were at the inner ring, and their axons were mainly located at the perifoveal ring, justifying the foveal morphology. The loss of GCs due to apoptosis and the neurodegeneration suffered by DM1 patients without DR suggests a functional dysfunction manifested by the decreased VA with low contrast demonstrated in other studies [4,6,14,26]. We correlated microperimetry values with the corresponding areas in the ETDRS grid although there is no exact location of the respective fields. We did not consider the displacement of the GCs from their respective fields due to the variability between subjects [27].

The GCL decrease was also reflected in the control group related with aging; this has already been demonstrated by other authors [26]. In the absence of differences between the mean ages of both groups, our findings are accounted for by the disease.

We found a generalized decrease in sensitivity in all quadrants in the DM1 group compared to the control group (Figure 4), including differences (*p* < 0.05) between groups in 3 out of the 4 quadrants in the 5° ring, in all 4 quadrants of the 3° ring and in 2 out of the 4 quadrants of the 1° ring. Significant differences were seen in the 3 rings in the temporal and inferior quadrants. A negative correlation was also seen between all sensitivity areas and patient age. Other studies have described the same phenomenon: decreased sensitivity was greater according to the earlier age at which they were found, and the severity of DR progressed [28,29].

Regarding the patient fixation values (location and stability) obtained with the MAIA, they were strongly associated with the patient’s visual function [28]. In Table 2, the fixation stability is similar between groups, showing that the macular sensitivity and the VA were not impaired. However, in DM1 patients, we observed a decreased macular integrity without differences compared to the control group. However, there were differences (*p* = 0.005) between groups in the average threshold, with lower values in the DM1 group probably related to the slight decrease in VA in this group. The fixation was also affected, with more fixation loss in DM1 patients (6.56%) than in the control group (0.42%) (*p* = 0.013).

In the anatomo-functional study, we found a negative correlation between VA and retinal sensitivity (Table 3) since good VA depends on correct retinal neuron function [30]. We found a decrease in GCs in the DM1 group; however, we cannot account for the loss of VA.

We also showed a negative correlation in the central ring between the retinal thickness and the retinal sensitivity (Table 4). These retinal changes are related to diminished retinal sensitivity [29] but were not significant for any of the three retinal studied layers. Furthermore, there was no decrease in retinal sensitivity in these patients.

Finally, only a significant negative correlation for the DM1 group was present (Table 4) in the GCL++ between the retinal sensitivity in the 5NI area and thickness in the TI area (−0.309 with *p* = 0.029). We did not find any other correlation between thickness and the macular sensitivity in the DM1 group. In general, we observed that the thickness changes in the different retinal layers caused a greater decrease in sensitivity in the temporal, inferior and nasal quadrants of the parafoveal ring.

All these results helped us to clarify the doubt about which of the mechanisms is the first one that triggers DR and whether it is a microvascular or neurodegenerative damage mechanism. Sensitivity changes without anatomical findings on structural OCT suggest that the second hypothesis is the most plausible option, as already postulated other studies [2,3,4,5,6,7,8,9,10,11,12,15,16,17,18].

Microperimetry is an excellent functional test complementary to VA and the retinal thickness measured with OCT. Thanks to the combination of these newly developed technologies, we can detect any type of retinal damage prior to the onset of DR, and we are able to treat affected patients before possibly irreversible complications have occurred.

## 5. Conclusions

In conclusion, aging and disease evolution in DM1 patients without DR signs generate a decrease in retinal sensitivity. There was a direct relationship between retinal sensitivity and macular thickness in the DM1 group.

## Figures and Tables

**Table 1:** Mean, standard deviation (SD) and statistical significance (*p*-value) of visual acuity (VA) in the LogMAR scale, spherical equivalent (SE) in diopters (D), axial length (AL) in mm and intraocular pressure (IOP) in mmHg between the control and type 1 diabetes mellitus (DM1) group. No statistical differences were found between groups (*p* > 0.05).

	Control Group		DM1 Group		*p*
	Mean	SD	Mean	SD	
VA (LogMAR)	−0.113	0.07	−0.130	0.01	0.910
SE	0.25	1.85	0.21	2.08	0.369
AL (mm)	23.61	2.14	23.37	1.16	0.297
IOP (mmHg)	16.85	2.47	16.59	3.00	0.150

**Table 2:** Mean and standard deviation (SD) of the rest of the parameters that the MAIA microperimeter offers for the control group and the DM1 group and a comparison between the values of both groups. The statistically significant values shown in bold and with an asterisk (*p* < 0.05). BCEA, Bivariate contour ellipse angle. The macular integrity and average threshold are measured in dB, the BCEA angle is measured in degrees, the area is measured in square degrees and fixation loses, P1, P2 are reported in %.

	Control Group		DM1 Group		*p*
	MEAN	SD	MEAN	SD	
Macular integrity index	47.35	28.64	46.59	35.20	0.990
Average threshold (dB)	28.16	1.46	26.44	3.46	0.005 *
Fixation stability P1 (%)	86.47	18.22	87.44	14.67	0.982
Fixation stability P2 (%)	95.70	7.43	96.64	4.91	0.492
BCEA 63 area (°^2^)	6.47	9.87	4.73	7.21	0.833
BCEA 63 angle (°)	9.36	62.36	2.01	60.84	0.477
BCEA 95 area (°^2^)	6.42	9.85	4.70	7.31	0.817
BCEA 95 angle (°)	9.36	62.36	1.52	61.19	0.468
Fixation losses (%)	0.42	2.83	6.56	12.99	0.013 *

**Table 3:** Spearman’s correlation between the retinal sensitivity measured by the MAIA microperimeter in the 9 ETDRS areas and the different parameters of visual function and age for the DM1 group. Statistically significant difference between groups (*p* < 0.05) are in bold. Correlation coefficient with *p* < 0.05 have one asterisk and with *p* < 0.01 have two asterisks. Nine ETDRS areas: SE, superior external; TE, temporal external; IE, inferior external; NE, nasal external; SI, superior internal; TI, temporal internal; II, inferior internal; NI, nasal internal; C-global, central; VA, visual acuity; SE, spherical equivalent; AL, axial length; and IOP, intraocular pressure.

Retinal Sensitivity									
	**5SI**	**5TI**	**5II**	**5NI**	**3SI**	**3TI**	**3II**	**3NI**	**0C**
**Age—correl coef.**	−0.413 **	−0.369 **	−0.493 **	−0.354 *	−0.455 **	−0.398 **	−0.364 **	−0.410 **	−0.361 **
**Sig.**	**0.003**	**0.008**	**<0.001**	**0.012**	**0.001**	**0.004**	**0.009**	**0.003**	**0.010**
**VA—correl coef.**	−0.356 *	−0.256	−0.301 *	−0.263	−0.341 *	−0.369 **	−0.341 *	−0.207	−0.195
**Sig.**	**0.011**	0.073	**0.034**	0.065	**0.015**	**0.008**	**0.015**	0.149	0.175
**SE—correl coef.**	−0.336 *	−0.262	−0.286 *	−0.204	−0.412 **	−0.371 **	−0.290 *	−0.232	−0.317 *
**Sig.**	**0.017**	0.066	**0.044**	0.155	**0.003**	**0.008**	**0.041**	0.105	**0.025**
**AL—correl coef.**	0.002	−0.053	−0.081	−0.121	−0.057	0.024	−0.048	−0.037	0.018
**Sig.**	0.989	0.715	0.574	0.404	0.693	0.869	0.739	0.801	0.903
**IOP—correl coef.**	0.314 *	0.239	0.205	0.193	0.267	0.300 *	0.146	0.293 *	0.276
**Sig.**	**0.027**	0.095	0.154	0.179	0.061	**0.034**	0.312	**0.039**	0.052

**Table 4:** Spearman’s correlation between the total retina (from the ILM to the BM line), GCL+ (from the RNFL to the IPL line) and GCL++ (from the ILM to the IPL line) thickness values in the central circle obtained by DRI Triton SS-OCT and the retinal sensitivity values obtained by the MAIA microperimeter in DM1 patients. Then, Spearman’s correlation between the parafoveal ETDRS areas (SI, TI, II and NI) of the total retina, GCL+ and GCL++ thickness layers measured by DRI Triton SS-OCT and the retinal sensitivity thresholds of the 3- and 5-degree radii measured by the MAIA microperimeter in the DM1 group. Statistically significant differences are in bold (sig. *p* < 0.05). The 9 ETDRS areas are as follows: C global, central global ring; SE, superior external; TE, temporal external; IE, inferior external; NE, nasal external; SI, superior internal; TI, temporal internal; II, inferior internal; and NI, nasal internal; correl coef, correlation coefficient; sig, statistically significant.

	ETDRS C (Retina)		ETDRS C (GCL+)	ETDRS C (GCL++)
**0C global—Correl coef**	−0.180		−0.070	−0.134
**Sig.**	0.210		0.627	0.353
**Retinal Sensitivity**	ETDRS SI (Retina)	ETDRS TI (Retina)	ETDRS II (Retina)	ETDRS NI (Retina)
**5SI—Correl coef**	0.085	−0.037	−0.031	0.011
**Sig.**	0.559	0.798	0.833	0.939
**5TI—Correl coef**	0.138	−0.022	0.016	0.055
**Sig.**	0.340	0.882	0.912	0.705
**5II—Correl coef**	0.013	−0.109	−0.104	−0.066
**Sig.**	0.931	0.453	0.472	0.649
**5NI—Correl coef**	0.022	−0.111	−0.122	−0.087
**Sig.**	0.882	0.443	0.398	0.550
**3SI—Correl coef**	0.087	−0.069	−0.067	−0.012
**Sig.**	0.546	0.634	0.643	0.934
**3TI—Correl coef**	0.131	−0.054	−0.010	0.051
**Sig.**	0.363	0.711	0.947	0.724
**3II—Correl coef**	0.105	−0.037	−0.031	−0.005
**Sig.**	0.470	0.800	0.829	0.970
**3NI—Correl coef**	0.026	0.024	−0.089	−0.060
**Sig.**	0.855	0.870	0.539	0.680
**Retinal Sensitivity**	ETDRS SI (GCL+)	ETDRS TI (GCL+)	ETDRS II (GCL+)	ETDRS NI (GCL+)
**5SI—Correl coef**	−0.002	0.051	−0.045	−0.082
**Sig.**	0.988	0.723	0.754	0.574
**5TI—Correl coef**	0.007	0.020	0.003	−0.048
**Sig.**	0.963	0.891	0.983	0.741
**5II—Correl coef**	−0.087	−0.052	−0.129	−0.148
**Sig.**	0.549	0.721	0.371	0.305
**5NI—Correl coef**	−0.122	−0.107	−0.184	−0.211
**Sig.**	0.399	0.459	0.201	0.140
**3SI—Correl coef**	−0.046	−0.031	−0.113	−0.107
**Sig.**	0.752	0.832	0.434	0.460
**3TI—Correl coef**	0.050	0.044	−0.037	−0.018
**Sig.**	0.729	0.760	0.801	0.901
**3II—Correl coef**	−0.038	−0.014	−0.087	−0.134
**Sig.**	0.794	0.922	0.550	0.352
**3NI—Correl coef**	−0.032	−0.053	−0.109	−0.142
**Sig.**	0.828	0.713	0.451	0.327
**Retinal Sensitivity**	ETDRS SI (GCL++)	ETDRS TI (GCL++)	ETDRS II (GCL++)	ETDRS NI (GCL++)
**5SI—Correl coef**	0.041	−0.104	−0.065	−0.132
**Sig.**	0.777	0.473	0.654	0.359
**5TI—Correl coef**	0.075	−0.102	−0.003	−0.055
**Sig.**	0.604	0.482	0.986	0.702
**5II—Correl coef**	−0.033	−0.258	−0.139	−0.201
**Sig.**	0.820	0.070	0.337	0.162
**5NI—Correl coef**	−0.062	−0.309	−0.208	−0.264
**Sig.**	0.670	**0.029**	0.148	0.064
**3SI—Correl coef**	0.026	−0.171	−0.105	−0.126
**Sig.**	0.855	0.234	0.467	0.383
**3TI—Correl coef**	0.119	−0.102	−0.017	−0.032
**Sig.**	0.412	0.480	0.907	0.823
**3II—Correl coef**	0.038	−0.163	−0.087	−0.156
**Sig.**	0.795	0.258	0.548	0.280
**3NI—Correl coef**	0.023	−0.232	−0.123	−0.184
**Sig.**	0.872	0.104	0.396	0.202

## Data Availability

The data presented in this study are available within the article.
